# Perspectives for the Use of CAR-T Cells for the Treatment of Multiple Myeloma

**DOI:** 10.3389/fimmu.2021.632937

**Published:** 2021-02-24

**Authors:** Marcin Jasiński, Grzegorz W. Basak, Wiesław W. Jedrzejczak

**Affiliations:** Department of Hematology, Transplantology and Internal Medicine, Medical University of Warsaw, Warsaw, Poland

**Keywords:** multiple myeloma, CAR-T cells, T lymphocyte, B-cell maturation antigen (BCMA), immunotherapy, cytokine release syndrome

## Abstract

During recent years considerable progress has been made in the treatment of multiple myeloma. However, despite the current improvements in the prognosis of this malignancy, it always ends with relapse, and therefore new therapy approaches for destroying resistant cancer cells are needed. Presently, there is great hope being placed in the use of immunotherapy against refractory/relapsed multiple myeloma which is unresponsive to any other currently known drugs. The most promising one is CAR-T cell therapy which has already shown tremendous success in treating other malignancies such as acute lymphoblastic leukaemia (ALL) and could potentially be administered to multiple myeloma patients. CAR-T cells equipped with receptors against BCMA (B-cell maturation antigen), which is a surface antigen that is highly expressed on malignant cells, are now of great interest in this field with significant results in clinical trials. Furthermore, CAR-T cells with other receptors and combinations of different strategies are being intensively studied. However, even with CAR-T cell therapy, the majority of patients eventually relapse, which is the greatest limitation of this therapy. Serious adverse events such as cytokine release syndrome or neurotoxicity should also be considered as possible side effects of CAR-T cell therapy. Here, we discuss the results of CAR-T cell therapy in the treatment of multiple myeloma, where we describe its main advantages and disadvantages. Additionally, we also describe the current results that have been obtained on using combinations of CAR-T cell therapies with other drugs for the treatment of multiple myeloma.

## Introduction

Multiple myeloma (MM) remains an incurable haematological malignancy although substantial progress in the treatment has occurred in recent years (1). The introduction of immunomodulatory drugs (IMiDs) such as thalidomide, lenalidomide, and pomalidomide has resulted in significant improvements for the long-term survival and the quality of life of MM patients (2, 3). The other drugs, namely proteasome inhibitors (bortezomib, carfilzomib, ixazomib), immune checkpoint inhibitors, and monoclonal antibodies have contributed substantially to improve the prognosis of MM patients (2, 4). Apart from these agents, another way of treatment is autologous stem-cell transplantation (ASCT), which is prescribed for patient populations with limited comorbidities and in good overall condition (5). Despite the progress made, MM generally progresses towards relapse/refractory to the available therapies (6). This creates an unmet need for new types of treatments, especially those which are based on a distinct mechanism of action. During recent years many new ideas have appeared and one of the most promising is chimeric antigen receptor T cell (CAR-T) therapy, which has already succeeded in the treatment of other haematological malignancies such as acute lymphoblastic leukaemia (ALL) and diffuse large B cell lymphoma and was approved by the FDA and EMA (7). In this review, our goal is to summarize the results, present challenges, and to describe new ideas for CAR-T cell treatment in refractory/relapsed multiple myeloma patients.

### Multiple Myeloma

Multiple myeloma (MM) is one of the most common malignancies of the hematopoietic system (15%) and accounts for approximately 1% of all neoplasms. Multiple myeloma typically affects elderly patients with a median age of 70 and with 63% of them being older than 65 (8). Although the prognosis in this disease is now much better than a few years ago, there are individual differences in survival that range from progression-free survival of more than 15 years in some patients (9), while approximately 15% of patients reach median survival of less than 2 years (10). The differences in survival outcomes occur because MM is a molecularly very heterogeneous disease (11). Additionally, the heterogeneity of this disease makes it improbable to develop a single molecularly targeted therapy (12). While progress can be made by searching for such a therapy for each distinct subset of MM, another way to approach the problem is to focus on cell surface targets that are common to all or a majority of MM cases. However, the main problem is that only a small fraction of antigens are common to myeloma cells while not being simultaneously expressed in other healthy tissues. Immunotherapies that include monoclonal antibodies, bi-specific T-cell engagers (BiTE), and CAR-T cell therapy are examples of such an approach with outstanding results in some cases.

### Chimeric Antigen Receptor T Cell Therapy

Chimeric antigen receptor T (CAR-T) cells are genetically engineered by the introduction of a construct coding for a receptor that is specific for target neoplastic cells. Hence, these cells are redirected to the selected target molecules on the surface of cancer cells. Specific CAR-T cell binding to targeted cancer cells triggers their death without major histocompatibility complex restriction (13). This approach provides the opportunity to overcome tumor escape mechanisms that can lead to cancer cell survival, such as i) the down-regulation of MHC molecules, ii) the processing of aberrant antigens, and iii) the creation of an immunosuppressive milieu which deactivates T cells (14). That being mentioned, it seems logical that this novel technique should find its application especially in tumors that rely on immunoregulatory mechanisms, and a good example of such malignancy is multiple myeloma.

CARs consist of four different elements: the recognition domain, the extracellular spacer, costimulatory elements, and the activating endodomain (15). The recognition domain [most common is a single-chain variable fragment—scFv (16)] binds to its ligand on the cell surface (signal 1), where the extracellular spacer provides the optimal space between the two cells to allow activation of the CAR-T cells and not suppression. Once binding to the ligand has occurred, the costimulatory molecule (CD28 or 4-1BB) becomes activated and initiates the response for signal 2; the onset of signal 2 is vital for optimal CAR-T cell activation and the prevention of anergy. The propagation of signals 1 and 2 are then transmitted to the activating domain (CD3-zeta) where both signals are forwarded to the CAR-T cell for activation (17).

**Figure 1:**
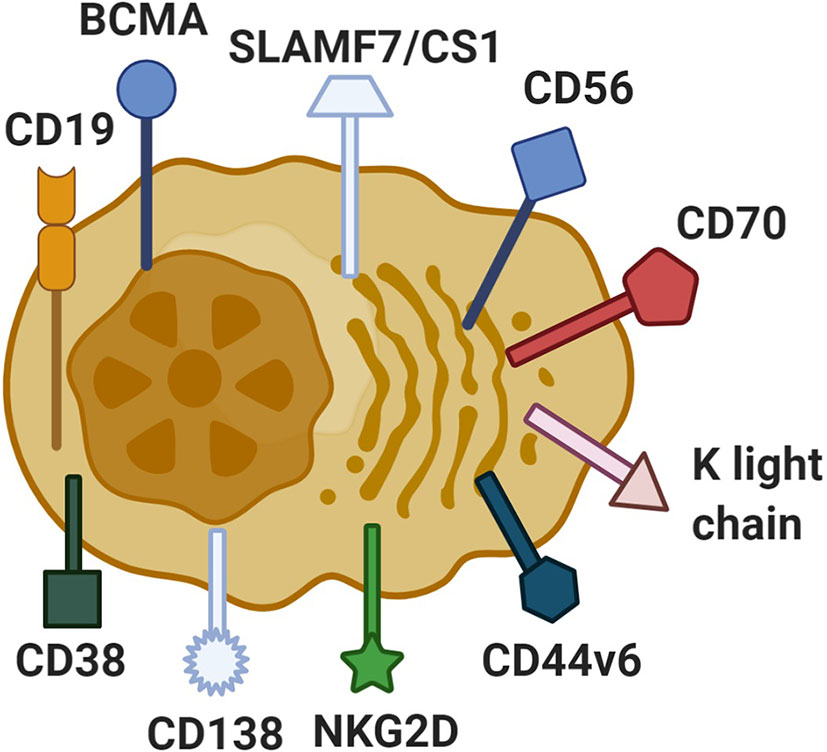
Antigens on multiple myeloma cells used as a target in CAR-T cell treatment. In the process of producing CAR-T cells, the main aim is to find the appropriate antigen which could be characterized by high expression levels on tumor cells and no expression on other healthy cells. The most known antigens used in the refractory/relapsed multiple myeloma CAR-T cell treatment are CD138, CD70, NKG2D, kappa light chain, CD19, SLAMF7/CS1, CD44v6, CD56, CD38, and BCMA. Created with BioRender.com.

The most important factor that determines the success of CAR-T therapy is the selection of target antigen. The first requirement in the process of designing CARs is to verify that the selected molecule is uniformly and sufficiently expressed on tumor cells. The second one is the absence or limited expression of the selected molecule on the normal cell surface. Otherwise, CAR-T cell therapy could lead to the debulking of the tumor but also to serious side effects with the deterioration of related normal cells (18). And that is exactly the main problem with multiple myeloma because it presents exceptional heterogeneity that effectively constricts the search for new antigens (19). However, some targets are now being used in clinical trials with promising results. One of them is CD19, with impressive results in the treatment of acute lymphoblastic leukaemia (20–23). Other antigens being under investigation are CD44v6, CD70, CD56, CD38, CD138, signaling lymphocyte-activating molecule F7 SLAMF7 (CS1), K light chain, NKG2D, and the most promising one about which we are particularly focused on in this review: B-cell maturation antigen (BCMA) (13, 15) (**Figure 1**).

The manufacturing of CAR-T cells involves the collection of patients’ T cells using apheresis as the first step. Once the T-cells are collected, they are further purified and activated with cytokines *in vitro*. Then they are transduced with viral vectors (retrovirus, lentivirus) containing a genetic construct coding for CAR. The efficiency of the transduction is in the range reaching up to 30% depending on the technique. After this, genetically engineered cells are further expanded, aliquoted, and stored frozen. In the meantime, the patient is receiving lymphocyte depleting chemotherapy to avoid rejection and enable the expansion of modified autologous CAR-T-cells. The procedure ends with the infusion of CAR-T cells to the patient in a specific dosing regimen (13).

## First-Generation B-Cell Maturation Antigen-Targeted Chimeric Antigen Receptor T Cell

As mentioned, the crucial problem in designing functional CAR-T cells is the selection of the appropriate antigen. Its expression on target cells should be uniform and preferably it should not be present on healthy cells. The problem is that the majority of antigens present on neoplastic cells are also expressed on some other normal cells.

The best known and explored target for CAR-T cells so far is CD19. This is an antigen of normal B cells that is also present on B cell leukemias and lymphomas (24, 25). But when it comes to multiple myeloma, which is also B cell neoplasia, CD19 is present only on a subset of malignant cells (26). Therefore, there is a risk that anti CD19 CAR-T cells would not eliminate myeloma, although it is also hypothesized that CD19+ component has myeloma stem cell functionality (27). Also, attempts to use anti-CD19 CAR-T cells in MM gave inconsistent results and will be reviewed later. This is why the search for other, more commonly expressed antigens on myeloma cells was undertaken. Among a few such candidates, B-cell maturation antigen (BCMA) exhibited the most favorable characteristics. Carpenter et al. showed that BCMA is present almost on all multiple myeloma cells and on none of 34 different tissues as well as on CD34+ hematopoietic stem cells (28). On the other hand, its expression was also found in normal plasma cells and mRNA for its production was noticed in the trachea and gastrointestinal organs which was proven to be associated with B cells in lamina propria and Peyer’s patches (28).

BCMA (CD269) is a member of the TNF receptor superfamily and is present on plasma cells and a small fraction of B cells (29). It binds B-cell activating factor (BAFF) or a proliferation-inducing ligand (APRIL) (30). Although it is particularly found on the cell surface, it can be also released into the serum when it is cleaved from the cell surface by *γ*-secretase. Patients with high infiltration of bone marrow with MM cells show elevated concentration levels of soluble BCMA in serum; this observation has proven to be particularly useful for the evaluation of CAR-T therapy (31).

Carpenter et al. conducted a few preclinical studies in which they have shown that transduced T cells demonstrated interesting features. CAR-T cells collected from patients could efficiently kill cells that possessed BCMA antigen on its surface even when soluble BCMA was added to the cells’ environment. These *in vitro* conditions reflect the bone marrow environment where BCMA is present on the cell surface and in the soluble form (28).

Human clinical trials on CAR-T cells in MM were first conducted with the same anti-BCMA CAR-T cells that were reported in the previous paragraph (28). Ali et al. selected 12 patients with a median of seven lines of previous treatments and administered to them increasing numbers of CAR-T cells 0.3 × 10^6^ cells/kg through 1 × 10^6^, 3 × 10^6^ up to the highest dose of 9 × 10^6^ cells/kg. The highest dose (9 × 10^6^ cells/kg) demonstrated the best clinical efficacy but also the most intensive adverse effects (32). All 12 patients experienced toxicities and each of them had cytopenias (mostly transient and attributed to conditioning therapy except in the case of two patients whose symptoms were protracted) that were easily managed. Another adverse event was cytokine release syndrome (CRS) with the greatest intensity in patients receiving the highest dose. CRS was characterized by fever, tachycardia, hypotension, hypoxemia, dyspnoea, and in one patient even with delirium. These symptoms were alleviated with the use of anti-IL 6 antibody tocilizumab and vasopressors. Some patients had elevated serum creatine phosphokinase levels with muscle weakness. Because of the BCMA presence on the normal plasma cells, the level of polyclonal serum antibodies dropped and some patients required intravenous immunoglobulin infusions to avoid infections (32). The best response achieved was with a patient whose bone marrow was 80% infiltrated with MM cells and for 28 weeks he had no detectable IgG lambda in the serum. Eventually, the patient relapsed which could be attributed to a small subset of MM cells which did not express BCMA on its surface and therefore were not selected by anti-BCMA CAR-T cell therapy (32). A few other interesting observations have been made after CAR-T cell infusion. Before CAR-T cell infusion the median CD8+/CD4+ T cell ratio was 1.1 but two weeks following the treatment CD8 positive cells became the main population of T cells. Moreover, these cytotoxic cells expressed new antigens such as PD-1 on their surface which are associated with senescence, exhaustion, and a decrease in proliferation ability. Summarizing, not only the loss or lack of antigen could contribute to eventual relapse but also loss of the efficient CAR-T cells (32).

In the continuation of this trial, Brudno et al. treated the next 24 patients with different dosages of CAR-T cells ranging from 0.3 × 10^6^ cells/kg, 1 × 10^6^, 3 × 10^6^ to 9 × 10^6^ cells/kg (33). Of the 10 patients who received 0.3–3 × 10^6^ cells/kg, only two showed partial or better responses to the treatment. Among the other 16 patients (two of them were treated a second time after the first clinical trial (32)) treated with 9 × 10^6^ cells/kg, the majority experienced partial or better responses but unfortunately the better the response was, the more intensive the adverse events were. Some of the responses exhibited symptoms of cytokine release syndrome (CRS) that was treated with tocilizumab, the others showed cytopenias with the most frequent being thrombocytopenia which was managed with the thrombopoetin agonist eltrombopag and prednisone. What was particularly interesting was the fact that patients with grades 3 and 4 of CRS also had higher numbers of MM cells in the bone marrow (33).

Moreover, the level of soluble serum BCMA decreased substantially after treatment with CAR-T cells, while during progression its concentration started to increase. This observation leads to the conclusion that soluble BCMA could be used as a predictive biomarker for CAR-T cell therapy response and also for early progression (33).

## Second-Generation B-Cell Maturation Antigen-Targeted Chimeric Antigen Receptor T Cell (Idecabtagene Vicleucel)

**Figure 2:**
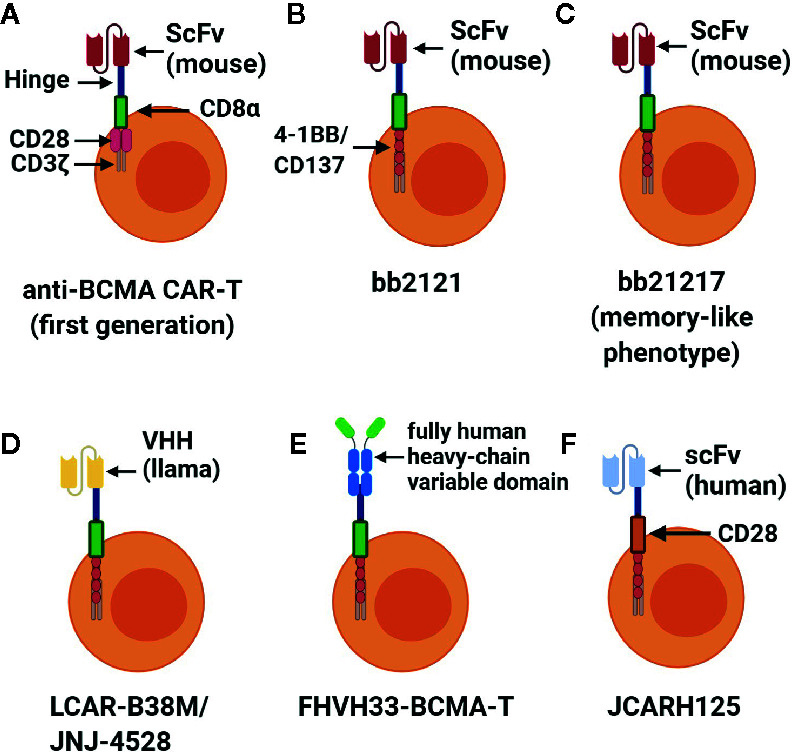
Different constructs of CARs targeting BCMA. BCMA is an antigen that is the most studied in CAR-T cells for relapsed/refractory MM. In use are the following types of anti-BCMA CAR-T cells **(A)** first generation of anti-BCMA CAR-T cells which consists of scFv as a binding molecule, hinge, transmembrane molecule (CD8α), costimulatory molecule (CD28), and an activating domain (CD3ζ); **(B)** the main change in the second generation of anti-BCMA CAR-T cells named bb2121 (Indecabtagene Vicleucel) was a new costimulatory molecule (4-1BB/CD137); **(C)** bb21217 differed from bb2121 with a memory-like phenotype of the former; **(D)** LCAR38M/JNJ-4528 are identical cells possessing single variable fragment of heavy chains (llama) instead of scFv (mouse) as a binding domain which can bind two different epitopes of BCMA; **(E)** FHVH33-BCMA-T as a binding domain was used with a fully human heavy-chain variable domain; **(F)** in the JCAR125 (Orvacabtagene Autoleucel) binding domain consists of a human scFv and a transmembrane domain costimulatory molecule (CD28). Created with BioRender.com.

After promising results in applying first-generation CAR-T cells, a search was initiated for determinants of better responses in MM patients. Several experiments have shown a significant positive correlation between peak CAR-T cell blood levels and clinical responses (34, 35). Furthermore, patients with better responses to treatment have been proven to have increased CAR-T cells that persist in the blood over a prolonged period of time (32, 33). Friedman et al. reasoned that the type of costimulatory molecule that is incorporated in the engineering of CAR-T cells could substantially affect both serum peak levels and persistence of CAR-T cells in blood. With that concept in mind, they designed T cells with chimeric antigen receptors but instead of CD28 as a costimulatory domain, as utilized in the previous preclinical and clinical experiments, they used another molecule—4-1BB (CD137) (36). Furthermore, an additional reason for changing the costimulatory domain was the high intensity and kinetics of CAR-T cell activation when CD28 is utilized. This high intensity and kinetics of CAR-T cell activation leads to the initiation of early-onset CRS. Moreover, T-cells with CD28 produced increased levels of Th-2 cytokines which mitigated their ability to kill cancer cells (37, 38) (**Figure 2**).

### bb2121

Raje et al. conducted a multicenter, open-label phase I trial with newly designed anti-BCMA CAR-T cells known as bb2121 which exhibited desired characteristics in preclinical experiments (39). They selected 33 patients with refractory/relapsed MM with a median age of 60 years and divided treatment into two phases. The first phase was with a dose escalation (50, 150, 450, and 800 × 10^6^ cells; 21 patients) in which the main aim was to evaluate the maximum tolerated dose (MTD). The second phase was with a dose expansion (150 to 450 × 10^6^ cells; 12 patients) where patients were treated with the estimated MTD to collect additional data (40). Both groups of patients were pre-treated with lymphodepleting therapy using fludarabine 30 mg/m^2^ and cyclophosphamide 300 mg/m^2^ over three days: –5, –4, and –3 prior to cell infusion. According to this and previous experiments, it seems unlikely that chemotherapy contributes to the outcomes because there were no responses in patients administered with subtherapeutic doses of anti-BCMA CAR-T cells (32). All but one patient received autologous stem cell transplantation previously (39).

The most frequent side effect of CAR-T cell treatment was cytopenia, especially neutropenia which occurred in 85% of patients and lasting a median of 10.9 months. Additionally, 75% of patients showed symptoms of CRS which occurred typically on the second day after infusion and persisted for 5 days on the average. Analysis of the severity of CRS showed a correlation between CRS score and the dose of the cells, CRP serum level, TNF alfa, ferritin level, and BCMA expression on MM cells. What is more, the peak presence of CAR-T cells in the blood was significantly higher in patients with a score of 3 and 4 on the scale of CRS. Twenty-one percent of patients received tocilizumab, and 12% were given corticosteroid to alleviate the symptoms of CRS. Furthermore, what is important is that these treatments did not affect CAR-T efficacy. Another side effect of the therapy was infections (42%), but they were easily controlled with antimicrobial agents (39).

The objective response rate (ORR) in all patients was 85%, and median PFS was 11.8 months. Forty-five percent achieved a complete response, and there was a significant effect of the dose of infused cells on the duration and frequency of response. Very good partial response (VGPR) occurred only in patients receiving at least 150 × 10^6^ CAR-T cells. Persistence of CAR-T in the host was improved compared to previous trials with 96, 86, 57, and 20% CAR-T cells after 1, 3, 6, and 12 months after infusion, respectively. In this study, 4-1BB instead of CD28 was used as a costimulatory domain. Another interesting remark was that the level of BCMA expression did not influence the patient’s response to CAR-T cell therapy (39). This could be because for anti-BCMA CAR-T cells only 222 BCMA molecules per cell were sufficient to induce cytotoxicity in *in vitro* experiments. Normally, MM cells exhibit far higher expression of this antigen on its surface (36).

While the main drawback of this trial was a small study group, it compared favourably to other salvage regimens for refractory and relapsed MM such as pomalidomide plus dexamethasone with a response of 31% (41) and BCMA directed antibody with a response of 60% (42).

During ASCO20 (American Society of Clinical Oncology Annual Meeting 2020), Munshi presented a multicenter, single-arm phase II trial with Indecabtagene Vicleucel for R/R MM. Enrolled patients (n = 140) were already pretreated with ≥3 prior regimens (Imbeds, PIs, anti-CD38 mAbs) and refractory to their last regimen per International Myeloma Working Group (IMWG) criteria (43). After leukapheresis, 88% of patients were administered with bridging therapy ≥14 days before lymphodepletion, which was conducted with cyclophosphamide 300 mg/m^2^ and fludarabine 30 mg/m^2^ on days −5, −4, and −3. Eventually, 128 patients received CAR-T cells and among them, four patients were treated with 150 × 10^6^ cells, 70 patients with 300 × 10^6^ cells, and 54 patients with 450 × 10^6^ cells. At data cut-off (16 Oct 2019) ORR was 73% and median PFS 8.6 months. Additionally, these values were significantly higher as the dose of CAR-T cells increased. Cytopenias occurred in 97% and CRS in 84% of patients but were most frequent within a group of higher CAR-T cell doses. Persistence and peak exposure was higher in responders when compared to non-responders (44).

### bb21217

There is evidence indicating that the persistence of CAR-T cells post-infusion is a determinant of the duration of the response. Other studies have shown that memory-like T cells represent a more potent and persistent phenotype of CAR-T cells compared to the unselected type. Therefore, enriching CAR-T cells in memory-like phenotype could lead to enhanced and prolonged responses. Such a change in phenotype of T cells may be reached by the utilization of phosphoinositide 3-kinase inhibitor bb007 during the *ex vivo* culture of CAR-T bb2121 cells. The product of this procedure named bb21217 after showing efficacy in preclinical studies was used in first-in-human study CRB-402 (45).

### LCAR-B38M

Zhao et al. designed dual epitope-binding CAR-T cells and utilized them in the first-in-human study named LEGEND-2, which was a multicenter on-label study conducted in China. LCAR-B38M cells have been created to bind to two different epitopes on BCMA target antigens on MM cells. This was intended to confer increased avidity of the cell binding to the target. Consequently, the dose of CAR-T cells could be lowered which was anticipated to decrease the frequency of the adverse events. Fifty-seven patients with refractory/relapsed MM and a median age of 54 were enrolled. All of them received conditioning therapy on days −5, −4, and −3 before infusion which consisted only of one agent—cyclophosphamide in a dose of 300 mg/m^2^ (the rationale for changing lymphodepletion protocol compared to other trials was safety). Also, the treatment regimen was distinct when compared to previous experiments. Patients having received a median dose of 0.5 × 10^6^ cells/kg split into three doses (20% + 30% + 50% of total dose over 7 days). ORR in this study was 88% (50 out of 57 patients exhibited a clinical response), CR occurred in 68% of patients, VGPR in 9%, and PR in 14%. The median duration of response at cut-off day was 14 months and PFS was 15 months. BCMA expression did not correlate with the response to the therapy. Although decreased doses of cells were administered, the adverse events occurred in the majority of patients. Ninety-one percent of patients developed pyrexia, 90% CRS, 49% thrombocytopenia, and 47% leukopenia. Stage 1 CRS occurred in 47%, stage 2 in 35% and stage 3 in 7% of patients. One patient developed grade 1 neurotoxicity: aphasia, agitation, and seizure-like behavior (46).

At the ASCO20 Meeting, Berdeja presented the results of the CARTITUDE-1 study with a median follow-up of 9 months when phase 1b was complete with n = 29. The CAR-T cells that were used were identical to LCAR-B38M cells used in the Chinese study. The median dose of the CAR-T cells was 0.73 ×10^6^ cells/kg administered in a singular infusion. The ORR for patients was 100%, where 76% had a stringent complete response. Further, 21% of the patients had VGPRs, and 3% had PR. This study showed that after 6 months 22/28 patients had a level of JNJ-4528 cells that was below the level of quantification, suggesting that the response did not correlate with CAR-T persistence in the peripheral blood (47, 48).

In another study, scientists investigated whether lymphodepletion therapy influences CAR-T cell function. They created anti-BCMA CAR-T cells with 4-1BB as a costimulatory domain and divided 25 patients into three cohorts as follows: cohort 1 with a range of infused cells: 1–5 × 10^8^ without lymphodepleting agents, cohort 2 with a range 1–5 × 10^7^ CART-BCMA cells preceded by cyclophosphamide 1.5 g/m^2^ and cohort 3 with a range 1–5 × 10^8^ CART-BCMA cells plus cyclophosphamide 1.5 g/m^2^. Percentage of responders in groups 1, 2, and 3 were as follows: 44, 20, and 64%. The longest response to therapy lasted for 32 months. Notable was the fact that BCMA expression on MM cells was significantly lower after CAR-T cell therapy (49).

### FHVH33-CD8BBZ

FHVH33-CD8BBZ designed by Lam et al. is a type of CAR-T cells which instead of a scFv possess a fully human heavy-chain variable domain (FHVH) which is connected to the 4-1BB costimulatory domain to increase the persistence of CAR-T cells in the host. The fully human heavy-chain variable domain is less immunogenic than scFv and the use of this receptor should lead to less severe adverse events. This could be especially advantageous in the case of a second transplantation. Moreover, genes used for FHVH creation are smaller than scFv which results in better expression of CARs on T cells. This feature could be particularly useful when it comes to designing CAR-T cells that target two antigens simultaneously (50). Mikkilineni et al. were conducting the first clinical trial of FHVH33-CD8BBZ with encouraging results. Twelve patients were divided into 3 groups with a different dosing regimen of 0.75, 1.5, and 3 × 10^6^ cells/kg. Ten patients out of 12 reached objective responses and CRS occurred in 11 patients. These results, while based on 12 patients only, have shown that the efficacy of CAR-T cells with FHVH could be reached at a far lower dosing regimen in comparison to products based on scFv (51).

### JCARH125

EVOLVE is an open-label phase I/II study in which a CAR-T cell product named Orvacabtagene Autoleucel (JCARH125) was administered to 62 patients. These CAR-T cells differed from other types of anti-BCMA CAR-T cells in that their binding domain is fully human with low affinity for soluble BCMA. In this study, patients with at least ≥3 prior therapies including Imbeds, PIs, ASCT, and anti-CD38 mAbs with lack of response to the last administered regimen were infused with 300 × 10^6^, 450 × 10^6^, or 600 × 10^6^ CAR-T cells. ORR was 92%, CR occurred in 36%, VGPR in 32% and PR in 24% of patients (52).

## Other Promising Target Antigens In Chimeric Antigen Receptor T Cell Therapy Against Multiple Myeloma

Taking into consideration that the main cause of relapse MM after CAR-T cell therapy is the loss of BCMA antigen, it is the basis of rationale to search for other antigens that could overcome this problem (53).

### SLAMF7/CS1

Signaling Lymphocytic Activation Family Member 7 (SLAMF7) is a robust marker of premalignant cells of MGUS and malignant cells in MM. The healthy cells that exhibit expression of SLAMF7 are: plasma cells, NK, and some CD8+ T cells, activated monocytes, and dendritic cells, and activated B cells (54, 55). This relative lack of specificity compared to BCMA requires changes in the treatment procedure. Therefore, SLAMF7 could be potentially used as a target of CAR-T cells only when associated with suicidal gene neighbouring CAR-T construct whose expression could be initiated on demand. An example of such a gene is inducible caspase 9 which is activated with rimiducid. This strategy aims to protect the patient from cytopenias in the case of severe adverse events (56). A functional antibody against SLAMF7 known as elotuzumab is already used in the treatment of MM, which confirms that it is a valuable target (57, 58). In a preclinical study, Wang et al. designed two CAR-T cell models. One targeted BCMA molecule whereas the second one was binding with SLAMF7. The effector function of both cells was similar although in a mouse model anti-SLAMF7 CAR-T cells proved to have better antitumor efficacy (59). Several SLAMF7-based CAR-T cells have entered clinical trial phases.

### CD19

Garfall et al. presented a study in which another type of CAR-T cell was used that uses CD19 as target molecule instead of BCMA. These cells are currently utilized in the management of acute lymphoblastic leukaemia with success (7, 21). The main difference between BCMA and CD19 is its profile of expression—CD19 was proposed to be present on the surface of myeloma stem cells whereas BCMA is present both on myeloma cells and normal plasma cells (60). The authors decided to infuse patients with a history of previous autologous stem cell transplantation (ASCT) with CAR-T cells expressing receptor targeting CD19 (CTL019) following high-dose melphalan conditioning therapy and second ASCT. Two out of 10 patients after ASCT + CTL019 compared with the first ASCT showed significantly longer PFS. The authors of this study indicate two possible reasons for such low efficacy of the therapy. First, due to safety reasons, the combination of CTL019 and ASCT required a 10-fold dose reduction of the former compared to other studies of CTL019. Therefore, the CAR-T cell engraftment within the patient’s bone marrow was impaired and consequently CD19+ cells were not sufficiently targeted. The second possible explanation is the inter-patient heterogeneity in the case of CD19+ expression on myeloma stem cells. Authors suspect that the immunophenotype of this subset of myeloma cells not can only vary between patients but also change over time in individuals (61) (**Table 1**).

**Table 1:** Results of clinical trials of CAR-T Cells in Multiple Myeloma.

Target antigen	Authors of the study	CAR	Number of patients	Conditioning therapy	Adverse events of CAR-T therapy	Effects of treatment
BCMA	Brudno (33)	CAR-T-BCMA	16	CXC 300 mg/m2 + FD 30 mg/m^2^	* Severe CRS (2) * Hypotension (6) * Adrenal insufficiency (4) * Encephalopathy (1) * Pancytopenia (2)	ORR 81% CR 13% VGPR 50%
BCMA	Raje (39)	B2121	33	CXC 300 mg/m^2^ + FD 30 mg/m^2^	* CRS (76%) * Neurologic toxicities (42%)	ORR 85% CR 45%
BCMA	Bardeja (45)	B21217	22	CXC 300 mg/m^2^ + FD 30 mg/m^2^	* CRS (59%) * Neurotoxicity (23%)	ORR 83%
BCMA	Zhao (46)	LCAR-B38M	57	CXC 300 mg/m^2^	* Pyrexia (91%) * CRS (90%) * Thrombocytopenia (49%) * Leukopenia (47%) * Neurotoxicity (2%) * AST increased 39%)	ORR 88% CR 68% VGPR 5%
BCMA	Madduri (48)	JNJ-4528	25	CXC 300 mg/m^2^ + FD 30 mg/m^2^	* CRS (88%) * Neutropenia (80%) * Anemia (76%) * Thrombocytopenia (72%)	ORR 91%* CR 29% VGPR 33%
BCMA	Cohen (49)	CAR-T-BCMA (human scFv)	25	CXC 1,5 g/m^2^ or without conditioning	* CRS (88%) * Neurotoxicity (32%)	Different ORR in 3 cohorts ranging 20-64%
BCMA	Mikkilineni (51)	FHVH-BCMA-T	12	CXC 300 mg/m^2^ + FD 30 mg/m^2^	* CRS (92%)	ORR 83% CR 17% VGPR 25%
CD19	Garfal (61)	CTL019+ASCT	10	MEL 140 mg/m^2^ or 200 mg/m^2^	* CRS (10%) * Autologous GVHD (10%) * Most of AEs are due to high-dose melphalan	ORR 80%

*21 patients evaluable for response.

CAR, chimeric antigen receptor; BCMA, B-cell maturation antigen; CRS, cytokine release syndrome; CXC, cyclophosphamide; FD, fludarabine; MEL, melphalan, ORR, overall response rate; CR, complete remission; VGPR, very good partial remission; AST, aspartate aminotransferase; GVHD, graft-versus-host disease; AE, adverse events; FHVH, fully-human heavy-chain-only; ASCT, autologous stem-cell transplantation.

## Bi-Specific Chimeric Antigen Receptor T Cells

*In vitro* experiments in which a mixture of two populations of CAR-T cells was administered (one population consisted of CD19 CAR-T cells and the second one of CD20 CAR-T cells) ended with unsatisfactory results. This is probably because of the different proliferation rates in these two populations which led to selective expansion of one population at the expense of the second one (62). One way to avoid competition between two populations of CAR-T cells is bi-specific CAR-T cells which express two receptors for two different target antigens on one T cell. In theory, it should allow CAR-T cells to debulk tumors containing both or one of the targeted molecules.

### B-Cell Maturation Antigen + Transmembrane Activator and Calcium-Modulator and Cyclophilin Ligand

A proliferation-inducing ligand (APRIL) is a natural ligand for both BCMA and transmembrane activator and calcium-modulator and cyclophilin ligand (TACI). According to Lee et al., TACI expression on human MM is limited to 78%, whereas BCMA was found on cells from all 50 tested patients. They have created bi-specific CAR-T cells which apart from BCMA targeted also TACI on MM cells. Reported results showed the efficacy of this approach in an *in vivo* model of tumor escape where BCMA+TACI− and BCMA−TACI+ cells were equally killed by CAR-T cells, whereas scFv bearing CAR-T cells against BCMA only was insufficient to prevent the outgrowth of BCMA-negative tumor (63).

### B-Cell Maturation Antigen + G Protein-Coupled Receptor Class C Group 5 Member D

G protein-coupled receptor class C group 5 member D (GPRC5D) was another antigen used for the creation of bi-specific CAR-T cells with BCMA. De Larrea et al. showed that in mice not only parallel infusion of anti-BCMA and anti-GPRC5D CAR-T cells could result in preventing tumor escape but also a single bicistronic vector encoding two 4-1BB-containing CARs has a significant efficacy and additionally avoids some problems with a parallel production of both CAR-T containing cells (64).

## Chimeric Antigen Receptor T Cell With Other Agents

### γ-Secretases Inhibitors

As mentioned before, the main obstacle that makes patients vulnerable to tumor relapse is the loss of the target antigen. A possible strategy to avoid at least some of the relapses could be the administration of inhibitors of *γ*-secretases, the enzymes responsible for BCMA loss from MM cell surface. Treatment of MM in NOD/SCID/gamma c−/− mice has a notable impact on the antitumor efficacy of anti-BCMA CAR-T cells alone. Furthermore, the antigen cleavage from the cell surface is decreased leading to less soluble antigen accumulation in the bone marrow which could improve antigen recognition by CAR-T cells and consequently MM cells being killed. The FDA-approved clinical trial (NCT03502577) has been initiated where gamma-secretase inhibitors are combined with anti-BCMA CAR-T cells (65).

### Lenalidomide

Wang et al. showed increased *in vitro* and *in vivo* effect of lenalidomide addition to genetically engineered T cells. Lenalidomide added during *in vitro* transduction and expansion of CAR-T cells increased cytotoxicity, memory maintenance, Th1 cytokine production, and immune synapse formation. Moreover, the addition of *in vivo* lenalidomide improved the persistence of anti-CS1 CAR-T cells in the host. These observations led to the design of a clinical trial combining lenalidomide and anti-CS1 CAR-T cells (59).

### PD-1 Inhibitor

Bernabei et al. demonstrated the efficacy of PD-1 inhibitor combination with anti-BCMA CAR-T cells. In the clinical study, they administered pembrolizumab, PD-1 inhibitor to five patients with relapsed MM after treatment with CAR-T cells. This combination proved to be efficient in inducing re-expansion of T cells in relapsed patients. However, re-expansion occurred infrequently and serious adverse events were evident (66).

## Summary And Future Directions

Previous studies have shown great efficacy of CAR-T cell therapy in MM in the context of remissions, even in a population of refractory/relapsed MM patients after many lines of treatment. It seems that the results of treatment are improving with the development of the new CAR constructs. Still, the main problem constitutes relapses of disease and shortness of remissions. Bearing in mind that relapses are frequently developed by MM cells possessing target antigens, it appears that future studies should be focused on improvements in the durability of response by prolonging the efficacy of CAR-T cells in the patient. That could be reached by modification of CAR constructs (especially in the costimulatory domain), use of different growth factors, and by avoiding CAR-T cell exhaustion (lower strength of binding to the antigen or decreasing intensiveness of activation signal; a combination of CAR-T cells with checkpoint inhibitors). Studies of CAR-T cells should be accompanied by studies of the biology of the cells that are responsible for relapses after CAR-T cell treatment. The study of the cells causing relapses can play a critical role in the search for new target antigens. Similar to transplantations, it seems that the combination of CAR-T cells with other drugs currently used in MM (new immunomodulatory drugs, proteasome inhibitors, or monoclonal antibodies) is the future of the field. Additionally, the conception of conditioning—CAR-T cell administration-consolidation/maintenance with new drugs or even a combination of CAR-T cell therapy with autologous/allogeneic transplantation requires deep consideration.

## Conclusions

Given the fact that MM is the second most frequent cancer in haematological practice, the need for new treatment strategies is considerable. The most promising trials include CAR-T cells targeted against BCMA antigen but use of other antigens like CS1 or CD19 could also lead to a better prognosis for refractory/relapsed MM patients. Although a few CAR-T cell products are currently registered by the FDA in the US for the treatment of other haematological malignancies, we are still waiting for the next phases of clinical trials regarding the use of CAR-T cells in multiple myeloma refractory/relapsed patients. Observations of the use of CAR-T cells in other malignancies have shown that a similar strategy will be probably common in the future in a matter of a few years. However, we should not forget about the potentially serious side effects of this therapy. Only selected MM patients can benefit from CAR-T treatment, and therefore, studies are needed to identify these groups. Furthermore, the majority of patients relapse after a relatively short time, so also studies regarding methods of blocking mechanisms of tumor escape are necessary (61) (**Table 2**).

**Table 2:** Advantages and disadvantages of CAR-T Cells therapy in Multiple Myeloma.

Advantages	Disadvantages
Novel therapy for refractory/relapsed patients	Potentially can cause life-threatening complications
Recognize cells without HLA expression	Relapses after treatment (antigen escape)
Eliminates only cells with targeted antigen	Immunogenicity of CARs
One-time treatment with long therapy-free intervals providing patients with a high quality of life	High costs of the therapy

## Author Contributions

All authors contributed to the article and approved the submitted version.

## Conflict of Interest

The authors declare that the research was conducted in the absence of any commercial or financial relationships that could be construed as a potential conflict of interest.
